# Bismuth in Gleet and Leucorrhœa

**Published:** 1860-07

**Authors:** 

**Affiliations:** Paris


### BISMUTH IN GLEET AND LEUCORRHŒA.

BY M. GABY, OF PARIS.

Repeated trials have convinced M. Gaby that injections of oxide of bismuth constitute the very best treatment of gleety discharges. Thirty parts are suspended in two hundred of rose water, and so injected as to leave as large a deposite of the salt as possible in the canal. Three injections per day should be employed at first, and then fewer. He has collected forty-three cases thus treated with success, five of which he briefly relates. Urethral discharges, unconnected with gonorrhœa, as observed in certain diatheses, in masturbation, venereal excesses, etc., and increasing in quantity, even after pure connection, have been successfully treated by this means in three instances.

Balanitis and balanoposthitis, and herpes præputialis yield rapidly to bismuth, applied in powder, after cleansing the part, and then covering with cotton. The various forms of vulvar leucorrhœa may be treated with bismuth. (One of these is entirely confined to the vulva, whether appearing as a consequence of follicular vulvitis, or without previous inflammatory symptoms. The latter is often met with in little girls.) Pregnancy, want of cleanliness, masterbation, worms, or contusion, are among the exciting causes. After removing all complication, the bismuth acts upon the discharge like a specific.

In the leucorrhœa of girls, powdering with bismuth is an excellent remedy. In ordinary vaginal leucorrhœa, occurring in women otherwise healthy, and having no other disease of the genito-urinary organs, the bismuth succeeds well.

Urethra-vaginal leucorrhœa, is almost always of infectious origin. In several instances reported, it yielded to bismuth after obstinately resisting other remedies. It is to be remembered that all the cases in which bismuth is useful are of the chronic description, and that pain and other signs of acute inflammation, contra-indicate its employment.—*North American Medical Reporter*.

